# Validation of repeated self-reported n-3 PUFA intake using serum phospholipid fatty acids as a biomarker in breast cancer patients during treatment

**DOI:** 10.1186/s12937-018-0402-6

**Published:** 2018-10-17

**Authors:** Sonja H. Brunvoll, Inger Thune, Hanne Frydenberg, Vidar G. Flote, Gro F. Bertheussen, Ellen Schlichting, Kristian S. Bjerve, Anette Hjartåker

**Affiliations:** 1 Department of Nutrition, Institute of Basic Medical Sciences, University of Oslo, PO Box 1046 Blindern, 0317 Oslo, Norway; 2 The Cancer Centre, Oslo University Hospital Ullevål, Oslo, Norway; 3 Department of Community Medicine, Faculty of Health Sciences, the Arctic University of Norway, Tromsø, Norway; 4 Department of Physical Medicine and Rehabilitation, St. Olav University Hospital, Trondheim, Norway; 5 Department of Neuromedicine and Movement Science, Norwegian University of Science and Technology, Trondheim, Norway; 6 Department of Breast and Endocrine Surgery, Oslo University Hospital, Oslo, Norway; 7 Department of Laboratory Medicine, St. Olav University Hospital, Trondheim, Norway; 8 Department of Clinical and Molecular Medicine, Norwegian University of Science and Technology, Trondheim, Norway

**Keywords:** Serum phospholipid fatty acids, Biomarker, Food frequency questionnaire, Pre-coded food diary, N-3 PUFAs, Fish, Validation, Breast cancer

## Abstract

**Background:**

The role of n-3 polyunsaturated fatty acids (PUFAs) in breast cancer is not clear and under debate. To explore this relationship it is important to have proper validated dietary assessment methods for measuring the intake of n-3 PUFAs. The aim of the current study is to validate two different methods used to assess the intake of selected n-3 PUFAs as well as food sources of long-chained n-3 PUFAs. Also, we aim to study how stable the intake of fatty acids is during breast cancer treatment.

**Methods:**

The study-population was patients with breast cancer (Stages I-II) or ductal carcinoma in situ (DCIS-grade III) undergoing treatment (*n* = 49) in Norway. Dietary intake was assessed by two self-administered methods, a 256 food item food frequency questionnaire (FFQ) and a 7-day pre-coded food diary (PFD). The FFQ was administered presurgery and twelve months postsurgery, and the PFD was administered shortly after surgery (10 +/− 2 days), six and twelve months postsurgery. Fasting blood samples (presurgery, six and twelve months postsurgery) were analysed for serum phospholipid fatty acids, a biomarker for intake of n-3 PUFAs.

**Results:**

Mean (SD) age was 54.2 (7.8) years at diagnosis, and the mean (SD) body mass index (BMI) was 24.8 (3.4) kg/m^2^. Correlation coefficients between dietary intakes of n-3 PUFAs measured with the FFQ and the PFD ranged from 0.35 to 0.66. The correlation coefficients between the PFD and the biomarker (serum phospholipid n-3 PUFAs) as well as between the FFQ and the biomarker demonstrated stronger correlations twelve months after surgery (*ρ* 0.40–0.56 and 0.36–0.53, respectively) compared to around surgery (*ρ* 0.08–0.20 and 0.28–0.38, respectively). The same pattern was observed for intake of fatty fish. The intake of n-3 PUFAs did not change during treatment assessed by the FFQ, PFD or biomarker.

**Conclusion:**

These results indicate that the FFQ and the PFD can be used to assess dietary intake of fish and n-3 PUFAs in breast cancer patients during breast cancer treatment. Still, the PFD shortly after surgery should be used with caution. The diet of patients undergoing breast cancer treatment was quite stable, and the intake of n-3 PUFAs did not change.

**Electronic supplementary material:**

The online version of this article (10.1186/s12937-018-0402-6) contains supplementary material, which is available to authorized users.

## Background

Knowledge of the role of individual fatty acids in health and disease is increasing, also when it comes to different types of cancers [1, 2]. The role of n-3 polyunsaturated fatty acids (PUFAs) in the diet of women diagnosed with breast cancer is not clear [3–5]. However, it has been proposed that a high intake of n-3 PUFAs prediagnosis, as well as postdiagnosis, may improve prognosis and survival after breast cancer [2, 6–9]. Being diagnosed with breast cancer may lead to changes in the diet, due to a desire to cure the disease, alleviate symptoms of nausea or to follow healthcare professionals’ advice [10].

To be able to investigate the n-3 PUFAs role in breast cancer prognosis or study dietary changes after diagnosis it is important to have proper methods to measure the dietary intake. Traditional methods for dietary assessment are usually based on self-report and include among other the food diary, which is usually conducted on several consecutive days. A food diary can provide detailed information about food intake, cooking methods, meals and eating frequency and can give relatively accurate information about dietary intake [11]. The weaknesses are that the tool is time-consuming both for the respondent and the investigator, multiple days are required to estimate habitual intake and the task of recording the diet may influence the dietary intake [11, 12]. The food frequency questionnaire (FFQ) on the other hand attempts to estimate usual dietary intake in one administration, is usually a lower burden on the respondent and the investigator and the cost of administration and handling is relatively low [13]. However, the FFQ is a closed method and may give fewer details on specific food types and cooking methods, and is more prone to measurement errors including recall errors as it is memory-dependent [13].

Biomarkers may be used as objective indicators on dietary intake and can be used to validate dietary assessment methods [14]. Using serum phospholipid fatty acids as biomarkers have been shown to be useful to reflect dietary intake of fatty acids [15]. However, no fatty acid biomarker can reflect the exact fat intake as it is subject to absorption and endogenous metabolism and can be affected by factors such as hormones [14–20]. Certain phospholipid fatty acids such as the long-chained n-3 PUFAs provide a better reflection of the dietary intake, while the saturated (SFAs) and monounsaturated fatty acids (MUFAs) provide a weaker estimate of the dietary content because of a high degree of endogenous metabolism [16, 17]. The serum phospholipid fatty acids are short- to medium-term biomarkers that reflect the dietary intake of the individual fatty acids for the last days or weeks [14, 16, 21].

It is important to have dietary assessment methods that are validated in the patient-group they are intended to be used. To our knowledge, methods assessing the intake of fatty acids in breast cancer patients during adjuvant breast cancer treatment have not previously been validated using a prospective method, a retrospective method and a biomarker. The aim of this study is to validate an FFQ and a pre-coded food diary (PFD) using serum phospholipid n-3 PUFAs as a biomarker for the intake of selected n-3 PUFAs as well as food-sources of long-chained n-3 PUFAs in breast cancer patients. By repeating our measurements over a year, we also examine how stable the intake of selected nutrients (including n-3 PUFAs) is during adjuvant breast cancer treatment.

## Methods

### Subjects and study design

A total of 60 women newly diagnosed with invasive histologically verified breast cancer (stages I-II) or ductal carcinoma in situ (DCIS-grade III) aged 35–75 years participated in a small clinical study between 2011 and 2013 at the Cancer Center, Oslo University Hospital Ullevål, Oslo; St.Olav University Hospital, Trondheim and Vestre Viken, Drammen, Norway. The patients were included before they underwent breast cancer surgery and were thereafter followed at the outpatient clinic. Patients with known severe illnesses (e.g. diabetes, heart disease) were excluded. A total of ten patients were excluded from the study after inclusion due to e.g. other diseases, unexpected settings in the family (e.g. death) or unable to participate due to their work setting (*n* = 50). One patient had incomplete dietary recordings, so finally 49 (82%) patients completed all the dietary assessment and had available blood samples at all points up to 12 months after surgery and were included in the present analyses. A total of 45 patients had breast cancer and 4 patients had DCIS-grade III.

### Patient characteristics and clinical measurements

The characteristics of the patients and clinical variables were assessed before any treatment (surgery, radiation, chemotherapy and/or endocrine therapy), including information regarding lifestyle habits, medical history and socioeconomic status. Anthropometric measurements were done with patients having no shoes on and wearing light clothes. Weight was measured to the nearest 0.1 kg on an electronic scale, height was measured to the nearest 0.5 cm and body mass index (BMI, kg/m^2^) was calculated. Waist circumference was measured 2.5 cm above the umbilicus; hip circumference was measured at the widest point of the buttocks. Waist-to-hip ratio was calculated by dividing waist circumference by hip circumference.

### Blood sample analyses

**Fig. 1:**
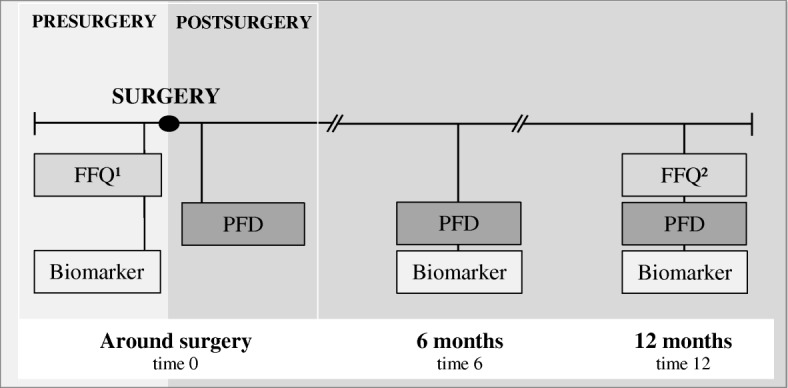
Timeline for FFQ, PFD and biomarker (serum phospholipid fatty acids). ^1^FFQ asks for the last year’s diet, filled in 2–8 days before surgery. ^2^FFQ asks for the last month’s diet. The PFD is filled in for seven consecutive days shortly after surgery (10 +/− 2 days after surgery), at six and twelve months postsurgery. The blood samples (biomarker) are drawn 2–8 days presurgery, at six and twelve months postsurgery

Fasting (overnight) venous blood samples were drawn presurgery (2–8 days before surgery), six and 12 months postsurgery (Fig. 1). The blood was drawn into serum clot activator tubes with separation gel and left to clot at room temperature for 30 min before centrifugation at 2000 g/rcf for 15 min. After centrifugation the serum was aliquoted into 1 ml cryotubes and left for 24 h at − 20 °C, before they were stored at − 80 °C. In February 2014 the samples were transferred to the Department of Laboratory Medicine, St. Olav University Hospital, Trondheim, for analyses. Serum phospholipid fatty acid concentrations were measured as described previously [22]. Plasma proteins were precipitated using 70% (*v*/v) perchloric acid (Merck) and total lipids extracted with n-butanol [23] using diheptadecanoyl-glycerophosphocholine and butylated hydroxytoluene (Sigma-Aldrich) as an internal standard and antioxidant, respectively. The phospholipids were isolated using Varian Bond Elute NH2 LRC columns and transmethylated in a N_2_ atmosphere using BF_3_ in methanol (Supelco Inc.) at a final concentration of 9.7% (*w*/w) at 135 °C for 30 min. The fatty acid methyl esters were extracted into isooctane before analyzed by GLC on a Hewlett-Packard 5890A using a 30 m SP2330 fused silica capillary column, 0.25 mm internal diameter, 0.20 μm film thickness (Supelco Inc.) with helium as a carrier at a linear gas velocity of 23 cm/sec. The initial column temperature was 170 °C programmed at 1.5 °C /min to 210 °C. The results were expressed as mg of phospholipid fatty acids per litre serum and recalculated to a percentage by weight (wt%) on the basis of twenty-two identified fatty acids (Total fatty acids). A normal human serum sample was included in each run to monitor analytical performance. The between-series CVs for 18:0, 18:1n-9, 18:2n-6, 20:3n-6, 20:4n-6, 20:5n-3, 22:6n-3 and Total fatty acids were 3.3%, 4.9%, 3.1%, 4.0%, 3.8%, 4.3%, 6.6% and 3.1% at a mean concentration of 169.5 mg/L, 117.7 mg/L, 214.5 mg/L, 18.1 mg/L, 120.0 mg/L, 68.7 mg/L, 121.8 mg/L and 1246.0 mg/L, respectively. The fatty acids examined in this study were the long-chained PUFAs EPA (eicosapentaenoic acid, 20:5n-3), DHA (docosahexaenoic acid, 22:6n-3) and the sum of the following n-3 PUFAs (hereafter termed “sum n-3”); ALA (alpha-linolenic acid, 18:3n-3), EPA, DPA (docosapentaenoic acid, 22:5n-3) and DHA.

### FFQ and PFD

Two different dietary assessment methods were used; an FFQ and a PFD. A modified version of an FFQ developed and validated by the Department of Nutrition, Institute of Basic Medical Sciences, University of Oslo, was used to gather data on the usual diet (retrospective data) [24–27]. The FFQ is 14 pages and includes 256 questions about food items arranged after the traditional meal pattern in Norway. Serving size per consumption is given in various household units such as spoons, deciliter, cups, glasses and slices and frequency of food items consumed range from never/seldom to several times a day. The types of fat used in cooking and as spread on bread are covered in separate sections, as is questions regarding dietary supplements usage, such as fish oil capsules and cod liver oil. At the end of the FFQ, there are open spaces where the patients can fill in food items or dietary supplements not covered in the questionnaire. Presurgery, the patients were asked to report their usual diet during the last year before they were diagnosed with breast cancer or DCIS-grade III. Twelve months after surgery the patients were asked to report their diet for the last month (Fig. 1). The questionnaires were the same, although covering different time-periods.

The patients also recorded all their food and drink consumption in a modified version of a PFD developed and validated by the Department of Nutrition, Institute of Basic Medical Sciences, University of Oslo [28–30]. The PFD is 19 pages and includes 310 questions on consumption of different food items arranged and grouped after the regular Norwegian meal pattern. Each food group has an open space at the end where food items/dishes and dietary supplements not covered in the pre-coded part can be listed. The design of the PFD is presented like a cross-table with time span at the top and type of food on the left side. Each day is divided into five time periods: 4 day periods where each is for 4 h (e.g. 6–10) and one night period from 22 to 06. Household units and photos from a validated photo-booklet were used to estimate the amounts consumed [31]. The photo-booklet contains 15 colour-photo series of different types of food, each with four serving sizes spanning from small to a large portion. The patients recorded the food they had eaten by filling in the type of food and the number of items eaten in the corresponding time period. The PFD was filled in for seven consecutive days shortly after surgery (10 +/− 2 days after surgery), 6 months postsurgery and 12 months postsurgery (that is 3 × 7 days, Fig. 1).

Both dietary assessment methods (FFQ and PFD) were obtained by self-report. However, all FFQs and PFDs were then manually checked by trained personnel; in case of inconsistencies or missing information the patients were contacted and the missing values were obtained. The completed FFQs and PFDs were scanned using the Cardiff TeleForm program version 10.5.1 (Datascan Oslo, Norway). Food and nutrient calculations, as well as fatty acid intake, were computerised using the food database AE-14 and the KBS calculation software system (**K**ost**b**eregning**s**ystem) at the Department of Nutrition, Institute of Basic Medical Sciences, University of Oslo. The food database AE-14 is based on the Norwegian food composition tables from 2014 and 2015 (http://www.norwegianfoodcomp.no/), supplemented with data from calculated recipes and other databases, and has a large number of fatty acids including n-3 PUFAs. The n-3 PUFAs that were investigated from the diet were the long-chained PUFAs EPA and DHA in addition to the total n-3 PUFAs. The “sum n-3”-variable (used for the biomarker; the sum of ALA, EPA, DPA and DHA) was not used for the diet as it was not considered relevant. The “sum n-3” variable accounts for approximately 80–90% of total n-3 PUFAs in the diet and hence the variable “total n-3” was used for diet. The food categories “fatty fish” (all fatty fish in the diet, e.g. salmon, trout, mackerel and herring) and “fish and fish products” (all fatty and lean fish as well as fish products, sushi and shellfish) were also examined.

### Statistical analyses

All analyses in this study are based on subjects who had completed all three assessments on all visits (PFD, FFQ and biomarker; *n* = 49). The sample size required when expecting a correlation coefficient around 0.40, with 80% statistical power and 5% significance level, is 46 subjects [32]. Statistical analyses were performed with IBM SPSS Statistics version 24.

Energy percent from macronutrients were calculated (E %). Median dietary intakes in the PFD and the FFQ, as well as median serum phospholipid fatty acids around surgery, 6 months and 12 months, were calculated and compared using Wilcoxon signed-rank test as the data distribution was skewed. The agreement between the methods was determined by cross-classification into tertiles. Spearman correlation coefficients were calculated between PFD and FFQ, PFD and biomarker (serum phospholipid fatty acids) and FFQ and biomarker. The 95% confidence intervals (CI) for the correlation coefficients were calculated using the Fisher Z method.

Spearman correlation coefficients were also calculated between the intake of EPA and DHA in the PFD/FFQ and the level of EPA and DHA in serum phospholipids, stratified by chemotherapy (yes/no), radiation (yes/no), endocrine therapy (yes/no), premenopausal/postmenopausal status, alcohol intake (median split) and BMI (<25/25≤). The Fisher’s Z transformation was used to investigate if there were any statistically significant differences in correlation coefficients between the strata. If any significant differences were detected between strata, Mann-Whitney test was performed to see if there were any differences in intake or in serum phospholipid fatty acids. A significance criterion of *p* < 0.05 was used.

## Results

Characteristics of the patients are presented in Table 1. The patients were on average 54.2 years at diagnosis (range 38–69), and their mean body mass index (BMI) was 24.8 kg/m^2^ (range 20.2–33.2). The majority of the patients received radiotherapy (38 patients, 78%), 30 patients (61%) received chemotherapy and 31 (63%) endocrine therapy.

**Table 1:** Descriptive statistics of the patients by means, SD and range, *n* = 49

Characteristics presurgery	Mean	SD	Range
Age at diagnosis, years	54.2	7.8	38–69
Education, years*	16.2	3.4	8–24
Postmenopausal, no (%)	32 (65)		
Smoking, no (%)	9 (18)		
Height, cm	168.1	5.9	155–181
Weight, kg	70.4	11.7	48.5–97.1
BMI, kg/m^2^	24.8	3.4	20.2–33.2
Waist circumference, cm	86.8	11.1	69.0–112.5
Waist/hip ratio	0.86	0.06	0.75–1.00
Treatment (around 6–12 months)			
Radiotherapy, no (%)	38 (78)		
Chemotherapy, no (%)	30 (61)		
Endocrine therapy, no (%)	31 (63)		

*SD* Standard deviation

*n = 48

The results from analysing serum phospholipid fatty acids are presented in Table 2. None of the fatty acids, expressed as wt% of total fatty acids in serum phospholipids, changed significantly over time. The full fatty acid composition is presented in the Additional file 1: Table S1.

**Table 2:** The biomarker serum phospholipid fatty acids in wt% and quartiles (P_25_‚ _75_), n = 49

Fatty acid, wt%	Time 0		Time 6		Time 12	
	Median	P_25_, _75_	Median	P_25_, _75_	Median	P_25_, _75_
Sum n-3^†^	10.24	8.03–11.54	9.33	8.07–11.12	9.31	7.99–10.85
EPA	2.54	1.84–3.17	2.23	1.56–2.79	2.19	1.53–3.03
DHA	6.24	5.44–7.13	6.10	5.13–6.73	5.84	4.97–6.72

wt%: Weight percent

time 0: 2–8 days presurgery, time 6: 6 months postsurgery, time 12: 12 months postsurgery

^†^Sum n-3: ALA (18:3n-3), EPA (20:5n-3), DPA (22:5n-3), DHA (22:6n-3)

### FFQ compared to PFD

The dietary intakes reported by the FFQ and PFD around surgery, at six and 12 months postsurgery are given in Table 3. When comparing dietary intake recorded around surgery, the median intake in the FFQ compared to the PFD was significantly higher for most nutrients, including total PUFA, total n-6 and n-3 PUFAs, EPA and DHA. When looking at E % from each nutrient most nutrients remained significantly higher in the FFQ, but total PUFA and total n-6 PUFA were no longer significantly different in the two methods. E % from total and saturated fat intake was higher in the PFD. The dietary intake at 12 months demonstrated a similar pattern as seen around surgery, with generally significantly higher intake measured with the FFQ including total PUFA, total n-6 and n-3 PUFAs, EPA and DHA and n-3 PUFAs from supplements. Looking at E % from each nutrient, most nutrients remained higher in the FFQ but there was no longer a significant difference in intake of total n-6 PUFAs and n-3 PUFAs from supplements. Also, E % from total and saturated fat was significantly higher in the PFD.

**Table 3:** Daily intakes by median, median E % and quartiles (P_25, 75_), *n* = 49

Dietary intake	PFD									FFQ					
	Time 0			Time 6			Time 12			Time 0			Time 12		
	E%	Median	P_25_, _75_	E%	Median	P_25_, _75_	E%	Median	P_25_, _75_	E%	Median	P_25_, _75_	E%	Median	P_25_, _75_
Energy, kJ		7557	6468–8834		6937	6119–8586		7419	6502–8071		8345*	7123–10,393		8254^†^	6746–9656
Carbohydrate, g	37	160	143–184	40	165	129–194	39	170	136–192	40*	197*	157–249	40^†^	196^†^	155–228
Sugar, g	7	28	19–41	5	25	14–45	6	25*	15–34	4*	20*	16–37	4^†^	16^†§^	9–31
Fibre, g		19	14–23		18	13–22		18	15–23		27*	20–31		27^†^	20–33
Protein, g	17	74	64–85	17	72	62–88	17	73	64–84	18	84*	70–106	17	79^†^	68–103
Alcohol, g	4	10	6–18	4	9	3–16	5	14	4–20	3	11	6–18	3^†^	7^†§^	3–15
Fat, g	39	77	68–94	36	70*	58–81	36	74	60–89	35*	82	64–100	35^†^	76	58–99
SFA, g	15	30	24–36	14	27*	21–31	14	28	22–34	12*	28	23–35	12^†^	27	19–33
PUFA, g	6	12	10–14	6	11	9–13	6	11	10–13	6	13*	11–18	6^†^	13^†^	11–17
Total n-6, g	4	9	7–11	4	8*	6–10	4	8	7–10	4	9*	7–13	4	9^†^	7–12
Total n-3, mg	1.3	3007	2309–3801	1.3	2941	1750–3368	1.3	2643	1713–4104	1.6*	3499*	2732–4841	1.5^†^	3544^†^	2499–4669
EPA, mg		372	160–605		358	148–519		403	171–605		472*	314–743		470^†^	318–645
DHA, mg		596	300–924		567	260–843		636	258–927		748*	500–1116		689^†^	478–1006
Fish, fish products, g		73	34–106		79	39–116		67^‡^	27–104		77	53–113		79	49–100
Fatty fish, g		21	4–42		16	4–37		26	4–46		21	13–40		20	13–30
Supplements, n (%)		30 (61)			26 (53)			29 (59)			34 (69)			30 (61)	
With n-3, n (%)		24 (49)			18 (37)			22 (45)			29 (59)			23 (47)	
Total n-3, mg ‖		628	356–1042		997	503–1215		595	300–1215		606	224–1368		619^†^	417–1711
EPA, mg ‖		163	76–198		180	121–202		133	92–198		124	54–3411		157	124–279
DHA, mg ‖		224	104–273		247	166–277		183	124–273		170	75–470		216	170–384

*E%* Energy percent, *PFD* pre-coded food diary, *FFQ* food frequency questionnaire

time 0: around surgery (FFQ: presurgery, PFD: postsurgery), time 6: 6 months postsurgery, time 12: 12 months postsurgery

EPA (20:5n-3), DHA (22:6n-3)

‖ within group taking n-3 supplements

* Significantly different from PFD time 0. The significance level is 0.05

^‡^ Significantly different from PFD time 6. The significance level is 0.05

^†^ Significantly different from PFD time 12. The significance level is 0.05

^§^ Significantly different from FFQ time 0. The significance level is 0.05

### Dietary changes

When studying each dietary assessment method separately, most nutrients did not change over time (Table 3). For the PFD the intake of total and saturated fat, as well as total n-6 PUFAs significantly decreased from shortly after surgery to 6 months postsurgery. The intake of sugar significantly decreased from shortly after surgery to 12 months postsurgery, and the intake of fish and fish products significantly decreased from 6 months to 12 months postsurgery. For the FFQ, the only significant change was a decreased intake of sugar and alcohol from the year before diagnosis to 12 months postsurgery.

### Fish intake

In the PFD, 46 patients shortly after surgery, 49 at six and 48 at 12 months postsurgery had eaten fish or fish products during the 7 days of diet-registration. The median daily intake of fish and fish products were 73, 79 and 67 g, respectively, of which 21, 16 and 26 g were fatty fish (Table 3). Around surgery, the Spearman correlation coefficients (Table 4) between intake of fish and fish products recorded by the PFD and the biomarker as well as fatty fish intake and biomarker, ranged from 0.14 to 0.25. At 6 months, the correlation coefficients between intake of fish and fish products and biomarker ranged from 0.24 to 0.33 and for fatty fish and biomarker from 0.15 to 0.27. At 12 months, the correlation coefficients between intake of fish and fish products and biomarker ranged from 0.16 to 0.27 and for fatty fish and biomarker from 0.30 to 0.40.

**Table 4:** Spearman correlation coefficients *(ρ)* between fish/fish products and fatty fish and biomarker, *n* = 49

Biomarker		PFD				FFQ			
		Fish and fish products		Fatty fish		Fish and fish products		Fatty fish	
	Time‡	*ρ*	95% CI	*ρ*	95% CI	*ρ*	95% CI	*ρ*	95% CI
Sum n-3†	0	0.19	−0.10, 0.45	0.21	−0.07, 0.47	0.03	− 0.25, 0.31	0.16	− 0.13, 0.42
	6	0.32*	0.04, 0.55	0.27	−0.01, 0.52				
	12	0.24	−0.04, 0.49	0.40*	0.14, 0.61	0.20	−0.09, 0.46	0.43*	0.17, 0.64
EPA	0	0.14	−0.15, 0.40	0.18	−0.11, 0.44	0.05	−0.23, 0.33	0.20	−0.09, 0.45
	6	0.24	−0.04, 0.49	0.15	−0.14, 0.41				
	12	0.16	−0.13, 0.42	0.39*	0.12, 0.60	0.13	−0.16, 0.39	0.31*	0.03, 0.54
DHA	0	0.25	−0.03, 0.50	0.23	−0.05, 0.48	0.04	−0.25, 0.32	0.22	−0.07, 0.47
	6	0.33*	0.05, 0.56	0.26	−0.03, 0.50				
	12	0.27	−0.02, 0.51	0.30*	0.02, 0.54	0.23	−0.06, 0.48	0.44*	0.19, 0.65

*CI* Confidence interval, *PFD* pre-coded food diary, *FFQ* food frequency questionnaire

†Sum n-3: ALA (18:3n-3), EPA (20:5n-3), DPA (22:5n-3), DHA (22:6n-3)

‡time 0: around surgery (FFQ: presurgery, PFD: postsurgery, biomarker: presurgery), time 6: 6 months postsurgery, time 12: 12 months postsurgery

* *p*<0.05

In the FFQ, the median daily intake of fish and fish products was 77 g the year before diagnosis and 79 g at 12 months, while intake of fatty fish was 21 and 20 g, respectively. All but one patient reported consuming fish or fish products the year before diagnosis and all patients reported consuming fish or fish products at 12 months. The Spearman correlation coefficients (Table 4) between intake of fish and fish products and biomarker presurgery ranged from 0.03 to 0.05, while fatty fish intake and biomarker ranged from 0.16 to 0.22. At 12 months the correlation coefficients for the intake of fish and fish products and biomarker ranged from 0.13 to 0.23 and for fatty fish intake and biomarker from 0.31 to 0.44.

### Supplements with n-3 PUFAs

About 70–85% of the supplements reported contained n-3 PUFAs and the supplements were a substantial contributor to total n-3 PUFAs, EPA and DHA intake in the current study. In the PFD, the number of patients taking supplements with n-3 PUFAs during diet-registration was 24, 18 and 22 shortly after surgery, at six- and 12 months postsurgery, respectively. The correlation coefficients between intake of total n-3 PUFAs, EPA and DHA from supplements and corresponding fatty acids in serum phospholipids around surgery ranged from 0.03 to 0.06. At six and 12 months the range was from 0.33 to 0.58 (Additional file 1: Table S2). In the FFQ, 29 patients reported taking supplements containing n-3 PUFAs the year before diagnosis and 23 reported taking it at 12 months. The correlation coefficients between total n-3 PUFAs, EPA and DHA from supplements and the corresponding fatty acids in serum phospholipids ranged from 0.34 to 0.54 at both time-points (See Additional file 1: Table S2).

### N-3 PUFAs

The Spearman correlation coefficients between dietary intake of n-3 PUFAs, EPA and DHA and the fatty acids in serum phospholipids are shown in Table 5. The correlations between the FFQ and the PFD ranged from 0.46 to 0.53 for total n-3 PUFAs, EPA and DHA around surgery and 12 months postsurgery. The Spearman correlation coefficients between the PFD and the biomarker around surgery ranged from 0.10 to 0.19, at 6 months the correlation coefficients ranged from 0.32 to 0.40 and at 12 months from 0.44 to 0.56. For the FFQ, Spearman correlation coefficients with the biomarker presurgery ranged from 0.28 to 0.33 and at 12 months it ranged from 0.38 to 0.51.

**Table 5:** Spearman correlation coefficients *(ρ)* and cross-classification for selected fatty acids, n = 49

Fatty acid		FFQ v. PFD				PFD v. Biomarker				FFQ v. Biomarker			
				Cross-classification				Cross-classification				Cross-classification	
	time‡	*ρ*	95% CI	ST	OT	*ρ*	95% CI	ST	OT	*ρ*	95% CI	ST	OT
n-3†	0	0.50*	0.26, 0.69	53	10	0.19	−0.09, 0.45	35	16	0.28*	0.00, 0.52	37	14
	6					0.40*	0.13, 0.61	45	14				
	12	0.51*	0.27, 0.69	47	8	0.56*	0.33, 0.73	57	6	0.50*	0.26, 0.69	47	8
EPA	0	0.53*	0.30, 0.71	57	6	0.10	−0.18, 0.37	35	16	0.30*	0.02, 0.54	45	14
	6					0.32*	0.04, 0.55	33	10				
	12	0.46*	0.20, 0.65	55	12	0.56*	0.33, 0.73	53	6	0.38*	0.11, 0.60	41	10
DHA	0	0.51*	0.27, 0.69	59	8	0.17	−0.12, 0.43	33	14	0.33*	0.05, 0.56	41	14
	6					0.38*	0.11, 0.60	59	12				
	12	0.48*	0.23, 0.67	47	12	0.44*	0.18, 0.64	51	8	0.51*	0.27, 0.69	55	8

*CI* Confidence interval, *PFD* pre-coded food diary, *FFQ* food frequency questionnaire

Cross-classification, % classified in ST: same tertile, OT: opposite tertile, grossly misclassified

†n-3: sum n-3 (ALA (18:3n-3), EPA (20:5n-3), DPA (22:5n-3), DHA (22:6n-3)) for biomarker and total n-3 PUFAs for PFD/FFQ

‡time 0: around surgery (FFQ: presurgery, PFD; postsurgery, biomarker; presurgery), time 6: 6 months postsurgery, time 12: 12 months postsurgery

* *p*<0.05

When studying E % from the n-3 PUFAs (Table 6) most of the correlation coefficients were not altered much, except for a stronger correlation coefficient for total n-3 PUFAs between FFQ and PFD around surgery (from 0.50 to 0.66) and a weaker correlation for EPA and DHA at 12 months (from 0.46 to 0.35 and from 0.48 to 0.38, respectively). The correlation coefficient for DHA at 12 months between FFQ (E %) and biomarker was also weaker than for crude intake (0.41 v. 0.51).

**Table 6:** Spearman correlation coefficients *(ρ)* and cross-classification for selected fatty acids, energy percent (E %) n = 49

Fatty acid		FFQ v. PFD				PFD v. Biomarker				FFQ v. Biomarker			
				Cross-classification				Cross-classification				Cross-classification	
	time‡	*ρ*	95% CI	ST	OT	*ρ*	95% CI	ST	OT	*ρ*	95% CI	ST	OT
n-3†	0	0.66*	0.47, 0.80	55	4	0.20	−0.08, 0.46	41	14	0.33*	0.06, 0.56	39	12
	6					0.39*	0.12, 0.60	45	12				
	12	0.48*	0.23, 0.67	57	10	0.52*	0.28, 0.70	49	6	0.53*	0.29, 0.70	57	10
EPA	0	0.58*	0.35, 0.74	51	4	0.08	−0.21, 0.35	31	16	0.35*	0.08, 0.58	49	10
	6					0.27	−0.01, 0.51	37	16				
	12	0.35*	0.08, 0.57	53	18	0.51*	0.26, 0.69	55	8	0.36*	0.09, 0.58	49	14
DHA	0	0.59*	0.36, 0.74	59	8	0.18	−0.11, 0.44	41	14	0.38*	0.11, 0.60	43	12
	6					0.39*	0.12, 0.60	53	14				
	12	0.38*	0.11, 0.60	47	16	0.40*	0.13, 0.61	45	10	0.41*	0.14, 0.62	43	8

*CI* Confidence interval, *PFD* pre-coded food diary, *FFQ* food frequency questionnaire

Cross-classification, % classified in ST: same tertile, OT: opposite tertile, grossly misclassified

†n-3: sum n-3 (ALA (18:3n-3), EPA (20:5n-3), DPA (22:5n-3), DHA (22:6n-3)) for biomarker and total n-3 PUFAs for PFD/FFQ

‡time 0: around surgery (FFQ: presurgery, PFD; postsurgery, biomarker; presurgery), time 6: 6 months postsurgery, time 12: 12 months postsurgery

* *p*<0.05

Cross-classification of patients by tertiles of reported intakes and serum phospholipid n-3 PUFA, EPA and DHA are shown in Tables 5 and 6. The percentages of patients categorized in the same tertile were generally higher at 12 months than around surgery, for PFD/biomarker 45–57% v. 31–41% and for FFQ/biomarker 41–57% v. 37–49%, respectively. Furthermore, the percentages of patients categorized in the opposite tertile, that is grossly misclassified, were generally lower at 12 months than around surgery, for PFD/biomarker 6–10% v. 14–16%, respectively. For FFQ/biomarker this pattern was not apparent with 10-14% grossly misclassified presurgery and 8-14% at 12 months postsurgery.

We also examined correlation coefficients according to patient characteristics (e.g. menopausal status or BMI), and treatment regime. The only significant difference was when stratified by chemotherapy (yes/no) for intake of EPA measured with the PFD and EPA in serum phospholipid at 6 months (*p* = 0.002). The Spearman correlation coefficient for the patients undergoing chemotherapy (*n* = 30) was *ρ* − 0.05 and for the patients not undergoing chemotherapy (*n* = 19) *ρ* 0.73. Mann-Whitney test comparing intake of EPA and EPA in serum phospholipids between the two strata demonstrated a significantly lower level of EPA in serum phospholipids in the patients undergoing chemotherapy (2.0 v. 2.6 wt%, *p* = 0.03) but no difference in intake of EPA.

## Discussion

In this study, we observed acceptable/moderate correlations between dietary intakes of total n-3 PUFAs, EPA and DHA measured with the FFQ and PFD. The correlations between the PFD and biomarker as well as between the FFQ and biomarker were stronger at 12 months postsurgery than around surgery. The same pattern was observed for fatty fish. Intake of most nutrients, including n-3 PUFAs, did not change during treatment assessed by the FFQ or PFD.

The median intakes of macronutrients were in general significantly higher in the FFQ than in the PFD, both around surgery and 12 months postsurgery. In comparison, other studies have demonstrated conflicting results when comparing an FFQ to a reference method. However, most often the FFQ gives higher estimates of macronutrient intake as well as intake of PUFAs [20, 26, 33–38]. A study among elderly (age 67–80 years) Norwegian women demonstrated a similar pattern as the present study with generally higher macronutrient intakes reported by a similar FFQ compared to weighed food records [26].

The intake of fish and fish products, as well as fatty fish, did not differ between the two dietary methods in the present study. Compared to the intake of fish and fish products reported by Norwegian women participating in the national Norkost 3 survey (age 18–70 years) the patients in our study reported around 20–40% higher fish intake (depending on time-point). However, fish intake in Norkost 3 is based on two 24 h recalls which might be too few administrations to give a good estimate on average fish-intake [39].

The intake of n-3 PUFAs among the patients in our study was higher than reported by others [6, 40]. The intake of total n-3 PUFAs was more than three times higher, and the intake of EPA and DHA was more than ten times higher than in an American study among newly diagnosed breast cancer patients [6]. This latter study is consistent with an other American study among women at risk for breast cancer [40]. Further, EPA and DHA in serum phospholipids were higher in patients in the present study compared to breast cancer patients in a Swedish study measured prediagnostic [41].

The correlations between intake of total n-3 PUFAs, EPA and DHA measured with the FFQ and PFD were acceptable/moderate and higher around surgery than at 12 months postsurgery. The correlations were within the same range or higher than demonstrated in validation studies comparing FFQ to dietary records, weighed food records or 24 h recalls in healthy adults [20, 42–44].

When comparing the PFD intake of total n-3 PUFAs, EPA and DHA with corresponding fatty acids in serum phospholipids, the timing of measurement was of great importance. It is clear that the correlation coefficients between PFD and serum phospholipid fatty acids were low around surgery, higher at 6 months and the highest correlation was observed at 12 months postsurgery. There might be several reasons for this pattern. At six and 12 months, the PFD was usually distributed the same day or one of the nearby days as the blood sampling, meaning that the biomarker represented the diet days/weeks before it was measured with the PFD. Around surgery, the blood sample was drawn 2–8 days before surgery, and the PFD was distributed 10 +/− 2 days postsurgery. Due to different timing, especially around surgery, the correlation coefficients observed might be underestimates of the true correlations. It is uncertain what kind of impact the breast cancer diagnosis or breast cancer surgery might have had on the actual diet around surgery or on filling in the quite extensive PFD. What is certain is that a breast cancer diagnosis can induce stress, and the level of stress might change from the time of diagnosis throughout the treatment period [45, 46]. It might be that our patients actually ate differently or changed their diet at the time of filling in the PFD compared to when the blood samples were drawn, although there were minimal changes throughout the year postsurgery. When it comes to filling in the PFD, it can be questioned if the workload of that task was too much, or that the patients could not put focus into it so soon after diagnosis. Also, repeated administrations of the PFD would pose a learning-effect in both the respondent and the study personnel throughout the study period.

With the FFQ, the timing of blood sampling and mapping of diet coincided better than for the PFD because of the retrospective nature of the FFQ. Both presurgery and 12 months postsurgery, the FFQ was distributed at the same time as the blood sample was drawn. The difference between the two time-points is that FFQ presurgery represented last year’s diet and at 12 months it represented the last month while the biomarker at both points represented the intake the last days or weeks. This might be an explanation for the stronger correlations seen at 12 months. Previously, studies have demonstrated that length of the reference period for the FFQ is of importance; the FFQs that report the diet the last month have somewhat higher correlations with the reference method in comparison to the ones reporting the diet the last year [47].

Even though the setting of our study was patients with breast cancer or DCIS-grade III undergoing treatment, the diet seemed quite stable. In particular, the dietary intake of total n-3 PUFAs, EPA and DHA in the FFQ and PFD did not change over the year. In the FFQ, only the intake of sugar and alcohol decreased from prediagnosis to 12 months postsurgery. The decrease in alcohol intake might have been due to the advice on restricting alcohol intake to a moderate level. On the contrary, such a decrease was not seen in the PFD and absolute intake of alcohol was also higher in the PFD at 12 months compared to FFQ, but no difference around surgery. We may question whether this difference in reporting of alcohol intake is a social desirability trait linked to the FFQ to a greater extent than to the PFD [48–51]. There were some changes in the diet from shortly after surgery to 6 months (decreased intake of total and saturated fat and total n-6 PUFAs) and from six to 12 months (decreased intake of fish and fish products). The changes around 6 months postsurgery might be because the time-point coincided with the time of most extensive treatment for most of the patients. It has previously been demonstrated that breast cancer patients going through chemotherapy have an overall lower energy intake compared to healthy women, characterized by lower intake of protein, fat and alcohol [52, 53]. On the contrary, chemotherapy has also been associated with an increased overall appetite and appetite for spicy and salty food [54]. Studies have further indicated that breast cancer patients are at risk for weight gain during and after chemotherapy, where both behavioural and physiological factors have been described to contribute to this weight gain [54, 55]. Approximately 30 to 60% of breast cancer patients have previously reported that they changed their eating habits after breast cancer diagnosis [10]. However, most studies are based upon data gathered a long time after diagnosis and by questionnaires asking about changes in the diet or by semi-quantitative FFQs recalling diet both before and after diagnosis [10].

The sum n-3 PUFAs, EPA and DHA in serum phospholipids did not change from prediagnosis to 12 months postdiagnosis and Lindberg et al. have previously demonstrated that the long-term tracking (over 3 years) of the biomarker (plasma phospholipid n-3 PUFAs) was highly significant [22]. However, there are several factors that can potentially influence the level of serum phospholipid fatty acids besides the dietary intake [56]. The presence of a breast tumour may possibly affect the fatty acid composition in serum phospholipids because lipid metabolism may be altered in breast cancer [57–59], but it is uncertain if and how it may influence our results. Also, both chemo- and hormonal therapy have been demonstrated to affect lipid metabolism [60, 61]. In this study, we stratified the patients according to treatment regime, to see if the treatment regime influenced the correlations between EPA/DHA intake and serum phospholipid fatty acids at any time point. The only significant difference found was the correlation between intake (PFD) and serum phospholipid EPA in the non-chemotherapy group (*ρ* 0.73) versus chemotherapy group (*ρ* − 0.05) at 6 months. The patients receiving chemotherapy had a significantly lower level of EPA in serum phospholipids, but there was no difference in the dietary intake. This single finding supports previous findings on chemotherapy and altered lipid metabolism [60, 61] and may indicate a limitation with our study. We also stratified the patients according to menopausal status, alcohol intake and BMI as both estrogen [16, 62], age [62, 63] and alcohol intake [64] have been suggested to influence serum phospholipid EPA/DHA, and obesity has been associated with altered lipid metabolism [59, 61]. However, we found no significant differences between the strata. Importantly, the number of patients in this study was low, and other possible findings might be underestimated due to lack of power. Further, there are not made any adjustments for the multiple testing performed in this study.

In the present study the PFD, FFQ and biomarker were compared to each other at each time-point separately to demonstrate the differences seen throughout the year after diagnosis. The validation approach included comparisons of the dietary assessment methods medians, the correlations between the methods as well as with the biomarker and cross-classification of consumption/concentrations into tertiles [65]. Because three methods were compared in this study, the method of triads could have been performed. However, due to small sample size and low correlation coefficients at some time-points, it was concluded that it would probably have generated several Heywood cases (the appearance of validity coefficients greater than one) and would not have given any additional information. The method of triads was therefore not conducted [66].

## Conclusion

The diet of patients with breast cancer or DCIS-grade III undergoing adjuvant treatment was quite stable and the intake of n-3 PUFAs did not change the year after surgery. However, our study suggests that the timing of mapping the diet is of importance. The FFQ and PFD in this study can be used to assess dietary intake of n-3 PUFAs and food sources of n-3 PUFAs in breast cancer patients during treatment, though the PFD shortly after surgery should be used with caution. This study demonstrates the importance of validating the dietary assessment tool within the patient-group it is intended to be used in as the patients’ situation and diagnosis may influence the choice of tool and usage.

## Additional file

Additional file 1: **Table S1.** The biomarker serum phospholipid fatty acids in wt% and quartiles (P_25_‚ _75_), *n* = 49. **Table S2.** Spearman correlation coefficients (*ρ*) for selected fatty acids from supplements, n = 49. (DOCX 55 kb)

## Abbreviations

ALA: Alpha-linolenic acid
BMI: Body mass index
CI: Confidence interval
DCIS: Ductal carcinoma in situ
DHA: Docosahexaenoic acid
DPA: Docosapentaenoic acid
E%: Energy percent
EPA: Eicosapentaenoic acid
FFQ: Food frequency questionnaire
KBS: Kostberegningsystem
MUFA: Monounsaturated fatty acid
OT: Opposite tertile
P_25_: Percentile 25
P_75_: Percentile 75
PFD: Pre-coded food diary
PUFA: Polyunsaturated fatty acid
SD: Standard deviation
SFA: Saturated fatty acid
ST: Same tertile
WT%: Weight percent
