# Supplementary material for: Validation of repeated self-reported n-3 PUFA intake using serum phospholipid fatty acids as a biomarker in breast cancer patients during treatment
Source: Nutr J. 2018 Oct 17;17:94. doi: 10.1186/s12937-018-0402-6 (PMC6192340; doi:10.1186/s12937-018-0402-6)
Supplement: Supplementary file 1 — Table S1. The biomarker serum phospholipid fatty acids in wt% and quartiles (P25‚ 75), n = 49. Table S2. Spearman correlation coefficients (ρ) for selected fatty acids from supplements, n = 49. (DOCX 55 kb) [file 12937_2018_402_MOESM1_ESM.docx]

**Supplementary Table 1** The biomarker serum phospholipid fatty acids in wt % and quartiles (P₂₅‚ ₇₅), n=49

|  | **time 0** | | **time 6** | | **time 12** | |
| --- | --- | --- | --- | --- | --- | --- |
|  | **wt %** | **mg/l** | **wt %** | **mg/l** | **wt %** | **mg/l** |
| **Fatty acid** | **Median** (P₂₅ˌ ₇₅) | **Median** (P₂₅ˌ ₇₅) | **Median** (P₂₅ˌ ₇₅) | **Median** (P₂₅ˌ ₇₅) | **Median** (P₂₅ˌ ₇₅) | **Median** (P₂₅ˌ ₇₅) |
| Total fatty acids^ǁ^ | **100** | **1310** (1169 – 1438) | **100** | **1330** (1190 – 1457) | **100** | **1354** (1183 – 1464) |
| 14:0 (myristic) | **0.42** (0.40 – 0.54) | **6.08** (4.83 – 7.26) | **0.48** (0.43 – 0.55) | **6.49** (5.54 – 7.33) | **0.48** (0.41 – 0.54) | **6.56** (5.25 – 7.60) |
| 16:0 (palmitic) | **28.03** (27.09 – 28.90) | **354.5** (322.7 – 409.8) | **28.00** (27.13 – 28.81) | **370.5** (330.6 – 416.4) | **27.96** (27.17 – 28.93) | **383.1** (337.6 – 422.1) |
| 18:0 (stearic) | **12.83** (12.16 – 13.75) | **164.6** (155.8 – 181.9) | **13.11** (12.28 – 13.90) | **171.6** (153.4 – 188.4) | **12.82** (11.89 – 13.79) | **173.1** (155.8 – 186.8) |
| 20:0 (arachidic) | **0.67** (0.60 – 0.74) | **8.56** (7.62 – 9.86) | **0.67** (0.59 – 0.77) | **8.65** (7.52 – 9.81) | **0.67** (0.62 – 0.76) | **8.93** (8.09 – 10.02) |
| 22:0 (behenic) | **1.25** (1.10 – 1.46) | **15.98** (13.83 – 20.00) | **1.27** (1.04 – 1.59) | **16.56** (13.73 – 19.96) | **1.30** (1.06 – 1.48) | **16.60** (14.00 – 19.67) |
| 24:0 (lignoceric) | **0.73** (0.62 – 0.88) | **9.85** (8.01 – 11.84) | **0.79** (0.62 – 0.99) | **9.59** (7.78 – 13.09) | **0.75** (0.59 – 0.84) | **9.89** (7.81 – 12.43) |
| 16:1 (palmitoleic) | **0.55** (0.40 – 0.75) | **7.01** (5.19 – 9.09) | **0.56** (0.46 – 0.71) | **7.85** (5.42 – 9.63) | **0.59** (0.50 – 0.75) | **7.79** (5.84 – 10.51) |
| 18:1 (oleic) | **10.29** (9.48 – 11.23) | **137.0** (116.5 – 156.3) | **10.34** (9.23 – 11.92) | **142.4** (119.6 -158.1) | **10.89** (9.38 – 11.99) | **143.6** (125.3 – 166.0) |
| 20:1 (eicosaenoic) | **0.16** (0.14 – 0.24) | **2.31** (1.75 – 3.25) | **0.18** (0.15 – 0.21) | **2.27** (1.95 – 2.89) | **0.19** (0.15 – 0.26) | **2.53** (2.02 – 3.61) |
| 22:1 (cetoleic) | **0.08** (0.05 – 0.12) | **1.06** (0.61 – 1.66) | **0.08** (0.05 – 0.11) | **0.98** (0.70 – 1.35) | **0.07** (0.05 – 0.10) | **0.98** (0.69 – 1.31) |
| 24:1 (nervonic) | **2.05** (1.87 – 2.31) | **27.89** (23.78 – 30.63) | **2.09** (1.77 – 2.53) | **28.00** (23.01 – 32.82) | **2.03** (1.83 – 2.39) | **27.23** (23.18 – 32.48) |
| 20:3n-9 (eicosatrienoic) | **0.11** (0.06 – 0.16) | **1.34** (0.79 – 2.14) | **0.13** (0.08 – 0.17) | **1.68** (1.11 – 2.24) | **0.13** (0.10 – 0.19) | **1.67** (1.27 – 2.59) |
| 18:2n-6 (linoleic) | **19.60** (17.70 – 22.23) | **265.7** (227.9 – 290.0) | **20.03** (17.11 – 22.25) | **267.2** (225.8 – 293.2) | **19.67** (17.97 – 21.76) | **265.4** (231.4 – 297.4) |
| 20:2n-6 (eicosadienoic) | **0.41** (0.37 – 0.45) | **5.29** (4.81 – 6.12) | **0.43** (0.39 – 0.49) | **5.60** (4.94 – 6.33) | **0.43** (0.40 – 0.47) | **5.86** (4.98 – 6.54) |
| 20:3n-6 (dihomo-γ-linolenic) | **2.19** (1.84 – 2.59) | **29.65** (23.92 – 35.30) | **2.58** (2.16 – 3.06) | **34.87** (27.05 – 38.86) | **2.60** (2.12 – 2.96) | **36.37** (28.09 -41.77) |
| 20:4n-6 (arachidonic) | **9.04** (7.67 – 10.74) | **119.1** (97.30 – 138.0) | **8.64** (7.43 – 10.15) | **116.1** (98.08 – 132.5) | **8.87** (7.35 -10.14) | **118.9** (97.1 – 142.3) |
| 22:4n-6 (adrenic) | **0.21** (0.18 – 0.25) | **2.74** (2.26 – 3.45) | **0.23** (0.18 – 0.26) | **2.98** (2.13 – 3.83) | **0.22** (0.18 – 0.26) | **2.80** (2.38 – 3.56) |
| 22:5n-6 (docosapentaenoic) | **0.11** (0.10 – 0.13) | **1.43** (1.26 – 1.70) | **0.13** (0.09 – 0.16) | **1.62** (1.14 – 2.17) | **0.12** (0.09 – 0.17) | **1.74** (1.25 – 2.30) |
| 18:3n-3 (α-linolenic) | **0.28** (0.20 – 0.34) | **3.68** (2.63 – 4.40) | **0.32** (0.22 – 0.40) | **4.10** (3.19 – 5.26) | **0.29** (0.23 – 0.42) | **4.05** (2.99 – 5.59) |
| 20:5n-3 (eicosapentaenoic) | **2.54** (1.84 – 3.17) | **30.62** (24.93 – 45.28) | **2.23** (1.56 – 2.79) | **29.88** (19.37 – 38.45) | **2.19** (1.53 – 3.03) | **28.84** (20.76 – 38.67) |
| 22:5n-3 (docosapentaenoic) | **0.99** (0.90 – 1.09) | **13.06** (11.00 – 15.32) | **0.98** (0.86 – 1.12) | **13.31** (10.78 – 15.51) | **0.97** (0.80 – 1.14) | **12.42** (11.18 – 15.30) |
| 22:6n-3 (docosahexaenoic) | **6.24** (5.44 – 7.13) | **81.96** (65.30 – 98.52) | **6.10** (5.13 – 6.73) | **78.41** (64.63 -90.09) | **5.84** (4.97 – 6.72) | **76.99** (66.33 – 92.22) |
| Sum SFA* | **44.45** (43.50 – 44.80) | **574.9** (523.2 – 640.0) | **44.33** (43.57 – 45.17) | **590.6** (526.3 – 643.3) | **44.25** (43.27 – 44.88) | **596.5** (523.2 – 656.2) |
| Sum MUFA† | **13.28** (12.66 – 14.24) | **179.6** (154.4 – 199.1) | **13.39** (12.14 – 15.09) | **180.0** (159.2 – 203.1) | **13.91** (12.50 – 14.77) | **186.4** (160.2 – 207.3) |
| Sum n-6^‡^ | **32.14** (30.37 – 33.98) | **422.8** (379.9 – 460.7) | **32.53** (30.54 – 33.77) | **422.2** (377.3 – 481.6) | **32.40** (30.44 – 33.98) | **437.4** (391.3 – 466.0) |
| Sum n-3^β^ | **10.24** (8.03 – 11.54) | **132.0** (106.7 – 157.3) | **9.33** (8.07 – 11.12) | **123.7** (105.0 – 146.3) | **9.31** (7.99 – 10.85) | **126.4** (107.7 – 143.9) |

wt %: Weight percent

time 0: 2-8 days presurgery, time 6: 6 months postsurgery, time 12: 12 months postsurgery

ǁ Total fatty acids: All 22 measured fatty acids serum phospholipids

*Sum SFA: 14:0 (myristic), 16:0 (palmitic), 18:0 (stearic), 20:0 (arachidic), 22:0 (behenic), 24:0 (lignoceric)

†Sum MUFA: 16:1 (palmitoleic), 18:1 (oleic), 20:1 (eicosaenoic), 22:1 (cetoleic), 24:1 (nervonic)

^‡^ Sum n-6: 18:2n-6 (linoleic), 20:2n-6 (eicosadienoic), 20:3n-6 (dihomo-γ-linolenic), 20:4n-6 (arachidonic), 22:4n-6 (adrenic), 22:5n-6 (docosapentaenoic)

^β^ Sum n-3: 18:3n-3 (α-linolenic), 20:5n-3 (eicosapentaenoic), 22:5n-3 (docosapentaenoic), 22:6n-3 (docosahexaenoic)

**Supplementary Table 2** Spearman correlation coefficients (*ρ*) for selected fatty acids from supplements, n=49

| **Fatty acid** |  | **FFQ v. PFD** | | **PFD v. Biomarker** | | **FFQ v. Biomarker** | |
| --- | --- | --- | --- | --- | --- | --- | --- |
|  | time‡ | *ρ* | 95% CI | *ρ* | 95% CI | *ρ* | 95% CI |
| n-3† | 0 | **0.64*** | 0.44, 0.78 | **0.05** | -0.24, 0.33 | **0.39*** | 0.12, 0.60 |
|  | 6 |  |  | **0.48*** | 0.23, 0.67 |  |  |
|  | 12 | **0.90*** | 0.84, 0.95 | **0.50*** | 0.26, 0.69 | **0.45*** | 0.19, 0.65 |
| EPA | 0 | **0.66*** | 0.46, 0.79 | **0.03** | -0.26,0.31 | **0.38*** | 0.11, 0.60 |
|  | 6 |  |  | **0.44*** | 0.18, 0.64 |  |  |
|  | 12 | **0.88*** | 0.80, 0.93 | **0.58*** | 0.36, 0.74 | **0.54*** | 0.30, 0.71 |
| DHA | 0 | **0.66*** | 0.47, 0.79 | **0.06** | -0.22, 0.34 | **0.37*** | 0.10, 0.59 |
|  | 6 |  |  | **0.37*** | 0.10, 0.59 |  |  |
|  | 12 | **0.91*** | 0.85, 0.96 | **0.33*** | 0.05, 0.56 | **0.34*** | 0.06, 0.57 |

CI: Confidence interval

PFD: pre-coded food diary, FFQ: food frequency questionnaire

†n-3: sum n-3 (ALA (18:3n-3), EPA (20:5n-3), DPA (22:5n-3), DHA (22:6n-3)) for biomarker and total n-3 PUFAs for PFD/FFQ

‡time 0: around surgery (FFQ: presurgery, PFD; postsurgery, biomarker; presurgery), time 6: 6 months postsurgery, time 12: 12 months postsurgery

* p˂0.05
